# New Books

**Published:** 2011-04

**Climate Change and Policy**

Gabriele Gramelsberger, Johann Feichter, eds.

New York:Springer, 2011. 240 pp. ISBN: 978-3-642-17699-9, $159

**Coping with Global Environmental Change, Disasters and Security**

H.G. Brauch, Ú. Oswald Spring, C. Mesjasz, J. Grin, P. Kameri-Mbote, B. Chourou, P. Dunay, J. Birkmann, eds.

New York:Springer, 2011. 1,815 pp. ISBN: 978-3-642-17775-0, $399

**Dealing with Contaminated Sites**

Frank A. Swartjes, ed.

New York:Springer, 2011. 1,114 pp. ISBN: 978-90-481-9756-9, $279

**Environmental Cardiology: Pollution and Heart Disease**

Aruni Bhatnagar, ed.

New York:Springer, 2011. 390 pp. ISBN: 978-1-84973-005-1. $199.95

**Global Change: Mankind–Marine Environment Interactions**

H.-J. Ceccaldi, I. Dekeyser, M. Girault, G. Stora, eds.

New York:Springer, 2011. 450 pp. ISBN: 978-90-481-8629-7, $179

**Handbook of Renewable Energy Technology**

Ahmed F. Zobaa, Ramesh C. Bansal, eds.

Hackensack, NJ:World Scientific, 2011. 876 pp. ISBN: 978-981-4289-06-1, $270

**Human Population: Its Influences on Biological Diversity**

Richard P. Cincotta, Larry J. Gorenflo, eds.

New York:Springer, 2011. 242 pp. ISBN: 978-3-642-16706-5, $129

**Implementing the New Biology: Decadal Challenges Linking Food, Energy, and the Environment**

Paula Tarnapol Whitacre, Adam P. Fagen, Jo L. Husbands, Frances E. Sharples, eds.

Washington, DC:National Academies Press, 2010. 52 pp. ISBN: 978-0-309-16194-7, $18.90

**In Search of Biohappiness: Biodiversity and Food, Health and Livelihood Security**

M.S. Swaminathan

Hackensack, NJ:World Scientific, 2011. 200 pp. ISBN: 978-981-4329-32-3, $88

**Intraseasonal Variability in the Atmosphere–Ocean Climate System, 2nd ed.**

William K.-M. Lau, Duane E. Waliser

New York:Springer, 2011. 320 pp. ISBN: 978-3-642-13913-0, $179

**Materials for Sustainable Energy**

Vincent Dusastre, ed.

Hackensack, NJ:World Scientific, 2010. 360 pp. ISBN: 978-981-4317-64-1, $148

**Pathways for Getting to Better Water Quality: The Citizen Effect**

Lois Wright Morton, Susan S. Brown, eds.

New York:Springer, 2011. 273 pp. ISBN: 978-1-4419-7281-1, $129

**Statistics for Earth and Environmental Scientists**

John Schuenemeyer, Larry Drew

Hoboken, NJ:John Wiley & Sons, Inc., 2011. 407 pp. ISBN: 978-0-470-58469-9, $110

**The Economic, Social and Political Elements of Climate Change**

Walter Leal Filho, ed.

New York:Springer, 2011. 875 pp. ISBN: 978-3-642-14775-3, $279

**The End of Energy: The Unmaking of America’s Environment, Security, and Independence**

Michael Graetz

Cambridge, MA:MIT Press, 2011. 384 pp. ISBN: 978-0-262-01567-7, $29.95

**The Energy Problem**

Richard S. Stein, Joseph Powers

Hackensack, NJ:World Scientific, 2011. 210 pp. ISBN: 978-981-4340-31-1, $38

**Understanding Knowledge as a Commons: From Theory to Practice**

Charlotte Hess, Elinor Ostrom, eds.

Cambridge, MA:MIT Press, 2011. 381 pp. ISBN: 978-0-262-51603-7, $20

**Urban Airborne Particulate Matter: Origin, Chemistry, Fate and Health Impacts**

Fathi Zereini, Clare L. S. Wiseman, eds.

New York:Springer, 2011. 656 pp. ISBN: 978-3-642-12277-4, $279

